# Supplementary material for: Asynchronous magnetic resonance elastography: Shear wave speed reconstruction using noise correlation of incoherent waves
Source: Magn Reson Med. 2022 Oct 27;89(3):990–1001. doi: 10.1002/mrm.29502 (PMC9792433; doi:10.1002/mrm.29502)
Supplement: Supplementary file 1 — DATA S1 MATLAB code used to generate the numerical simulations of Figure 1 [file MRM-89-990-s002.zip › k-Wave/helpfiles/gradientSpect.html]

gradientSpect :: Functions (k-Wave)


# gradientSpect

Calculate the gradient using a Fourier spectral method.

## Syntax

```
fx = gradient(f, dx)
fx = gradient(f, dx, [], deriv_order)
fn = gradient(f, dn, dim)
fn = gradient(f, dn, dim, deriv_order)
[fx, fy] = gradient(f, dn)
[fx, fy] = gradient(f, dn, [], deriv_order)
[fx, fy, fz, ...] = gradient(f, dn)
[fx, fy, fz, ...] = gradient(f, dn, [], deriv_order)
```

## Description

`gradientSpect` calculates the gradient of an n-dimensional input matrix using the Fourier collocation spectral method. The gradient for singleton dimensions is returned as 0.

A 1D example:

```
% compute gradient of a 2 period sinusoid
x = pi/20:pi/20:4*pi;
y = sin(x);
dydx = gradientSpect(y, pi/20);

% plot gradient and error compared to analytical solution
subplot(2, 1, 1);
plot(x, cos(x), 'k-', x, dydx, 'bx');
axis tight;
title('dy/dx');
subplot(2, 1, 2);
plot(x, cos(x) - dydx, 'k-');
axis tight;
title('Relative Error');
```

A modification of the example given with the MATLAB `gradient` function (x and y are reversed):

```
% generate input and compute gradient
[x, y] = meshgrid(-2:.2:2, -2:.2:2);
z = x .* exp(-x.^2 - y.^2);
[px, py] = gradientSpect(z, [.2, .2]);

% plot
figure;
contour(z)
hold on;
quiver(py, px);
hold off;
```

## Inputs

|  |  |
| --- | --- |
| `f` | matrix or vector to find the gradient of |
| `dn` | array of values for the grid point spacing in each dimension. If a value for `dim` is given, `dn` is the spacing in dimension `dim`. |

## Optional Inputs

|  |  |
| --- | --- |
| `dim` | optional input to specify a single dimension over which to compute the gradient for n-dimension input functions |
| `deriv_order` | order of the derivative to compute, e.g., use 1 to compute df/dx, 2 to compute df^2/dx^2, etc. (default = 1) |

## Outputs

|  |  |
| --- | --- |
| `fx, fy, ...` | gradient in the each dimension, where x corresponds to dim = 1, y corresponds to dim = 2 etc. |

## See Also

`getFDMatrix`, `gradient`, `gradientFD`
